# Crosstalk among proximal tubular cells, macrophages, and fibroblasts in acute kidney injury: single-cell profiling from the perspective of ferroptosis

**DOI:** 10.1007/s13577-024-01072-z

**Published:** 2024-05-16

**Authors:** Yulin Wang, Ziyan Shen, Shaocong Mo, Han Zhang, Jing Chen, Cheng Zhu, Shiqi Lv, Di Zhang, Xinhui Huang, Yulu Gu, Xixi Yu, Xiaoqiang Ding, Xiaoyan Zhang

**Affiliations:** 1 Department of Nephrology, Zhongshan Hospital, Fudan University, No. 180 Fenglin Road, Shanghai, 200032 China; 2 Shanghai Medical Center of Kidney Disease, No. 180 Fenglin Road, Shanghai, 200032 China; 3 Shanghai Key Laboratory of Kidney and Blood Purification, No. 180 Fenglin Road, Shanghai, 200032 China; 4 Shanghai Institute of Kidney and Dialysis, No. 180 Fenglin Road, Shanghai, 200032 China; 5 Department of Digestive Diseases, Huashan Hospital, Fudan University, Shanghai, 200040 China; 6 Division of Nephrology, The Affiliated Changzhou No.2 People’s Hospital of Nanjing Medical University, Changzhou, 213100 Jiangsu China; 7 Department of Nephrology, Union Hospital, Tongji Medical College, Huazhong University of Science and Technology, Wuhan, 430022 China

**Keywords:** AKI, Ferroptosis, Single-cell analysis, NMF, Crosstalk

## Abstract

The link between ferroptosis, a form of cell death mediated by iron and acute kidney injury (AKI) is recently gaining widespread attention. However, the mechanism of the crosstalk between cells in the pathogenesis and progression of acute kidney injury remains unexplored. In our research, we performed a non-negative matrix decomposition (NMF) algorithm on acute kidney injury single-cell RNA sequencing data based specifically focusing in ferroptosis-associated genes. Through a combination with pseudo-time analysis, cell–cell interaction analysis and SCENIC analysis, we discovered that proximal tubular cells, macrophages, and fibroblasts all showed associations with ferroptosis in different pathways and at various time. This involvement influenced cellular functions, enhancing cellular communication and activating multiple transcription factors. In addition, analyzing bulk expression profiles and marker genes of newly defined ferroptosis subtypes of cells, we have identified crucial cell subtypes, including Egr1 + PTC-C1, Jun + PTC-C3, Cxcl2 + Mac-C1 and Egr1 + Fib-C1. All these subtypes which were found in AKI mice kidneys and played significantly distinct roles from those of normal mice. Moreover, we verified the differential expression of Egr1, Jun, and Cxcl2 in the IRI mouse model and acute kidney injury human samples. Finally, our research presented a novel analysis of the crosstalk of proximal tubular cells, macrophages and fibroblasts in acute kidney injury targeting ferroptosis, therefore, contributing to better understanding the acute kidney injury pathogenesis, self-repairment and acute kidney injury-chronic kidney disease (AKI-CKD) progression.

**Supplementary Information:**

The online version contains supplementary material available at 10.1007/s13577-024-01072-z.

## Introduction

Ferroptosis is an iron-dependent regulatory necrotic subroutine characterized by aggregation of iron ions and increased lipid peroxidation [1]. Ferroptosis plays a very important role in a wide range of both physiological and pathological processes, including tumors, acute kidney injury (AKI), neurodegenerative diseases, and ischemia–reperfusion injury [2]. Interestingly, among all organs, the kidney appears to be more vulnerable to iron-dependent ferroptosis. Conducted research on this matter indicate that inactivation of the ferroptosis regulator glutathione peroxidase 4 (GPX4) can directly induce acute renal failure by triggering iron toxicity in mouse renal tubular cells [3]. Not coincidentally, in an oxalate-induced AKI model, it has also been demonstrated that ferroptosis can directly lead to tubular necrosis [4]. With progression and advancements in research, new ferroptosis regulatory pathways and critical proteins including FPS1 and DHODH have been gradually discovered in recent years. All these advancements provide new ideas and targets for the treatment of many diseases such as tumors, liver diseases and COVID-19 [5–7]. Therefore, investigating ferroptosis in AKI is important to deepen the understanding of disease mechanisms and developing therapeutic strategies.

In recent years, significant advancements have been achieved in the cellular and molecular levels of AKI. In addition, the recent maturation of single cell sequencing technology has significantly impacted the field of AKI [8, 9]. An increasing number of studies have revealed the temporal and spatial distribution of cells in AKI, introducing the concept of an immune microenvironment for AKI. Despite all these advances, the pathogenesis of AKI at the cellular and molecular mechanisms is still not completely understood. The kidney generally is recognized having a very distinct microenvironment where a several numbers of cells including epithelial cells, endothelial cells, fibroblasts, vascular smooth muscle cells, resident immune cells and infiltrating immune cells engage in a highly diverse interactions [10]. Simultaneously, the development of AKI affects a variety of cells in the kidney to varying degrees [11], making the cross-talk among various cell types a focal point of discussions in the recent years. Research studies in animal models of ischemia reperfusion injury induced AKI (IRI-AKI) have shown that in the early stages of AKI, tubular cell injury first triggers an innate immune response [12]. Subsequently, tubular epithelial cell injury, activation of renal resident immune cells and aggregation of different subpopulations of infiltrating immune cells occur, with almost all immune cells involved in the progression of AKI, contributing to aseptic inflammation of the kidney [13]. Meanwhile, activation of fibroblasts emerges as a pivotal mechanism leading to the transformation of AKI into chronic kidney disease (CKD) [14]. During the AKI, a crucial role is played by the inflammation processes. Initially, they adopt at the pro-inflammatory phenotype in the early stages of injury, while changing to an anti-inflammatory phenotype during the repair phase, counteracting the effects of abnormal inflammation and supporting renal tubular regeneration [10]. The critical role of fibroblasts in facilitating the transformation and progression of AKI to CKD has been widely recognized [15]. Yet, research focused on fibroblasts, macrophages, and renal tubular epithelial cells and ferroptosis in AKI remain limited. In recent years, the significance role of ferroptosis in the development and progression of AKI has been increasingly recognized and emphasized [16]. To delve deeper into this area, we have employed single-cell analysis techniques to examine cellular interactions, using both a database focusing on ferroptosis and single-cell sequencing data.

In our research, we explored the cell-to-cell interactions between proximal tubular cells (PTC), macrophages and fibroblasts in murine AKI kidneys based on the single-cell dataset GSE139506. We applied single-cell non-negative matrix factorization (NMF) for both clustering and analysis, identifying subgroups of proximal tubular cells, macrophages and fibroblasts with ferroptosis-related genes. We proceeded with conducting further analysis on the communication between every identified subtype.

## Materials and methods

### Data acquisition and processing

We acquired the single-cell RNA sequencing data and clinical information of GSE139506 from the Gene Expression Omnibus (GEO) database (http://www.ncbi.nlm.nih.gov/geo). Applying the Seurat pipeline to the single-cell sequencing data we processed the Seurat objects constructed by GSE139506 by normalizing, scaling and dimensional reduction for subsequent analysis. Normal kidney samples were not involved in this study and only AKI mice models were included. We annotated cell types in the Seurat objects using commonly recognized marker genes [17]. We downloaded expression profiling for GSE34351 by array and related clinical information from the GEO database. We obtained profiles of ferroptosis-related genes from FerrDb (http://www.zhounan.org/ferrdb). Meanwhile a comprehensive database containing 784 ferroptosis-related articles was extracted from the PubMed database (https://www.ncbi.nlm.nih.gov/pubmed) on regulators and markers of ferroptosis and related diseases. In total, 253 regulators (including 108 drivers, 69 suppressors, 35 inducers and 41 inhibitors), 111 markers and 95 diseases associated with iron dystrophy were identified [18]. After duplication removal, a total of 205 genes was obtained for further analysis.

### Single-cell non-negative matrix factorization (NMF)

NMF is a well-known dimensionality reduction method that can decompose a non-negative matrix into the product of a non-negative basis matrix and a non-negative coefficient matrix [19]. Unlike principal component analysis (PCA) [20], NMF’s unique features makes it especially useful in many fields such as artificial intelligence [21], biological data mining [22], and image processing [23] specifically due to its non-negativity constraint [19]. We began filtering single-cell Seurat objects based on ferroptosis-related genes. Cells lacking expressions any ferroptosis-associated genes as well as ferroptosis-associated genes that were not expressed in all cells were completely excluded from analysis. We applied the method of NMF using snmf/r setting, allowing for identification of up to 10 clusters [24, 25]. These results were incorporated into Seurat objects for further dimensional reduction analysis. This process yielded in several different clusters of a certain class of cells. In the next step we used, the FindAllMarker software package to filter the signature genes for each NMF cell cluster. For each cluster, if it contained ferroptosis-related genes with log2 (Fold Change) (logFC) > 1, the cluster was labeled “gene + cell type” by selecting the ferroptosis-related gene with the largest LogFC, otherwise, the cluster was labeled “non-ferroptosis–cell type”.

### Pseudo-time analysis

We utilized Monocle2 package (http://cole-trapnell-lab.github.io/monocle-release) of R to conduct pseudo-time analysis in the single-cell Seurat objects, with samples both in AKI and sham groups [26]. Essentially, Monocle2 uses an algorithm to learn that certain gene expression sequences during each cell’s development must undergo changes in gene expression sequences, and thus determine whether each cell is at an early or late stage of development [27]. This analysis allows the expression of ferroptosis-related genes at various stages of cell development to be determined and displayed in a heatmap.

### Cell-to-cell crosstalk analysis

We analyzed intercellular communication using the CellChat package [28], which determines intercellular interactions by assessing the expression of ligand and receptor pairs within a cell population [29, 30]. In principle, CellChat applies network analysis and pattern recognition methods to predict the signal inputs and outputs of cells and how these signals regulate cellular function, and thus assess intercellular crosstalk [31].

### Transcription factor analysis

We conducted transcription analysis using SCENIC (Single-Cell Regulatory Network Inference and Clustering) software package (http://scenic.aertslab.org) [32]. This approach allows us to leverage genomic regulatory codes to pinpoint transcription factors and cell states. SCENIC is developed on this concept particularly to map out gene regulatory networks and classify cell states simultaneously. [33]. The regulatory subnetworks generated by SCENIC analysis are scored as a whole, not just TFs or individual genes, so this approach is robust to drop-outs [34].

### Functional enrichment and differentially expressed gene analysis

Functional enrichment analysis is done based on the gene ontology (GO) database, a cornerstone resource in the field of bioinformatics used for enrichment analysis and consists of three main components: cellular component, molecular function, and biological process [35].Differentially expressed genes extracted from GSE34351 expressing profiling were screened by applying the limma software package [36]. Genes were primarily considered different if met the following criteria (|log2 Foldchange| ≥ 1, *p* value < 0.05).

### Animals

The findings from our single-cell analysis results were further validated using IRI mice models. We selected C57BL/6 male mice at 6–8 weeks of age. Mice were induced with bilateral ischemia–reperfusion injury (IRI) model by bilateral traumatic renal stalk clamping for 27 min at a controlled body temperature of 36–37 °C [32]. Kidney samples were harvested and analyzed 24 h after IRI and verified by Real-Time Quantitative PCR. For control comparisons, we used kidney samples harvested from sham mice of the same strain and age.

### Real-time quantitative PCR

To validate the expression changes at the tissue level of genes identified by single-cell RNA-seq using real-time quantitative PCR (RT-qPCR). The kidney tissues were treated with TriZol reagent (Sigma) to extract tissue-derived RNA. The extracted total RNA went under reverse transcription-PCR and real-time quantitative PCR after treatment with PrimeScript RT Master Mix Kit (TaKaRa) and SYBR Premix Ex Taq Kit (TaKaRa). We used the 2^ − ΔΔCt^ method to calculate the relative mRNA expression of each gene using GAPDH as an internal reference. The specific primers used are listed in Table 1.

**Table 1:** The primers for real-time qPCR

	Forward	Reverse
Gapdh	AGGTCGGTGTGAACGGATTTG	GGGGTCGTTGATGGCAACA
Egr1	TCGGCTCCTTTCCTCACTCA	CTCATAGGGTTGTTCGCTCGG
Jun	TTCCTCCAGTCCGAGAGCG	TGAGAAGGTCCGAGTTCTTGG
Cxcl2	CCAACCACCAGGCTACAGG	GCGTCACACTCAAGCTCTG

### Multiplex immunofluorescence

To initiate immunofluorescence (IF) staining, we started with epitope retrieval by autoclaving the sections with target retrieval solution (DAKO, S1699). Following this process, sections underwent permeabilization in 0.1% Triton X-PBS and blocking using 2.5% horse serum. We incubated sections overnight at 4 °C with specified primary antibodies. The primary antibodies included anti-AQP1 (from Abcam, UK, 1:250), anti-Egr1 (from Abcam, UK, 1:100), anti-Jun (from Abcam, UK, 1:500), anti-FAP (from Abmart, China, 1:200), anti-CD68 (from Cell Signaling Technology, USA, 1:100) and anti-CXCL2 (from Proteintech, USA, 1:100). The next day, we applied a mixture of fluorescent-labeled secondary antibodies along with DAPI for nuclear staining, incubating them at room temperature for 45 min and sent for further analysis. Subsequently, the initiate immunofluorescence staining results were observed under a confocal microscope to determine the presence of a subpopulation of cells by examining the colocalization of fluorescence.

### Statistical analysis

For comparisons between constant variables, we used the *t*-test, with a significance level of 0.05. All statistical analyses were performed using R version 4.1.3.

## Results

**Fig. 1:**
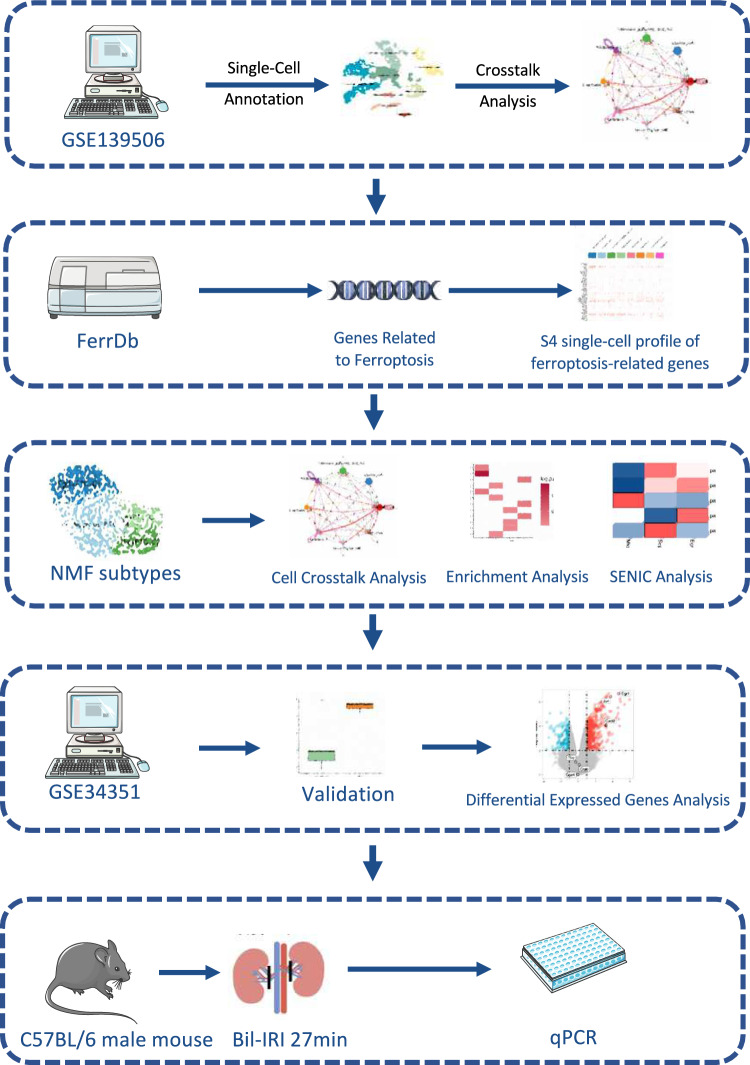
The flowchart of the study

The workflow of the study is presented in Fig. 1.

### Cell types, distribution, interactions, and ferroptosis-related genes in AKI mice by single-cell analysis

**Fig. 2:**
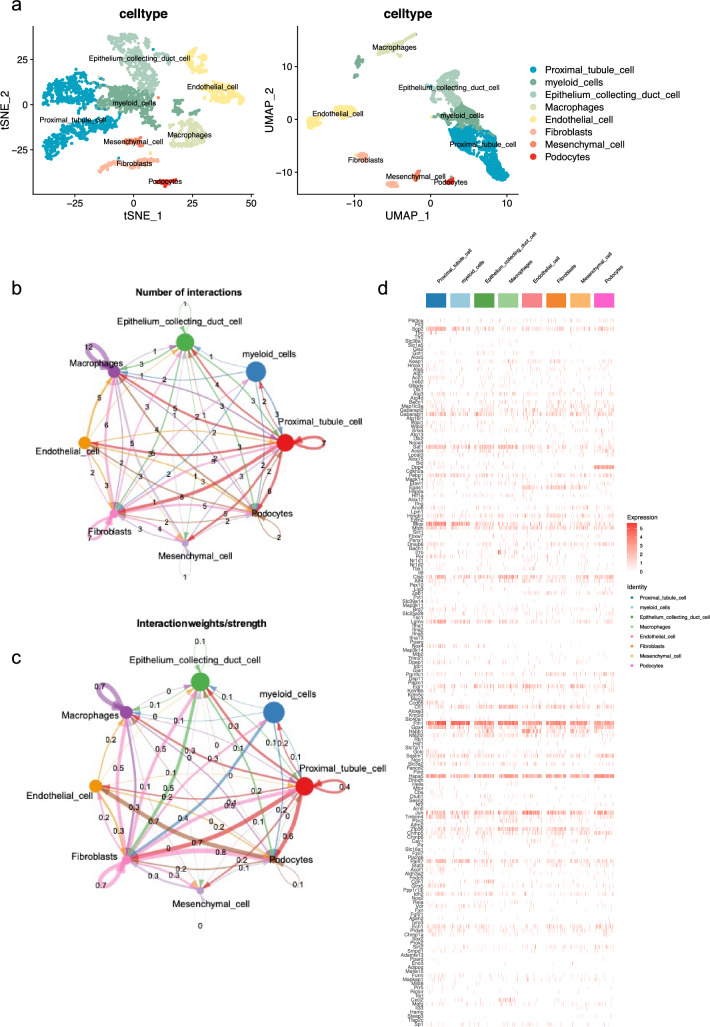
Cell types, distribution, interactions, and ferroptosis-related genes in AKI mice by single-cell analysis based on GSE139506. **a** Annotated tSNE and UMAP plots of all cell types in GSE139506, containing 8 categories. Only samples of AKI were contained in the analysis. **b**, **c,** respectively, show the numbers and weights of the crosstalk pathways among all types of cells from GSE139506. **d** Heatmap of expression levels of 205 ferroptosis-related genes in all types of cells in GSE139506

Single cell analysis of IRI mice model leveraging the dataset from GES139506 identified eight cell types in kidneys of AKI mice, including proximal tubule cells, myeloid cells, epithelium collecting duct cells, macrophages, endothelial cells, fibroblasts, mesenchymal cells, and podocytes. The distribution of these cell types was depicted in t-distributed stochastic neighbor embedding (tSNE) plots and uniform manifold approximation and projection (UMAP) plots by Dimplot function (Fig. 2a). Figure 2b demonstrated the number of cell-to-cell interactions pathways between these eight identified cell types, including 13 interactions between proximal tubule cells and fibroblasts, and 8 interactions between proximal tubule cells and macrophages, and 8 interactions between macrophages and fibroblasts. The intensity of these cell-to cell interactions was illustrated in Fig. 2c.

The heatmap in Fig. 2d presented the gene expression patterns of 205 ferroptosis-related genes across different cell types in AKI mouse kidneys. Genes such as Egr1, Hspa5, and Mtdh were expressed in all 8 types of cells highlighting their potential broad involvement in the ferroptosis process within the kidney. Specifically, proximal tubule cells showed a higher expression of genes such as Lgmn, Miox, Fth1, and Gpx4 suggesting a unique role of these cells in ferroptosis-driven processes. On the other hand, genes such as Ctsb and Cxcl2 had the highest expression in macrophages. Fibroblasts were characterized by enhanced expression of genes such as Pgrmc1 and Epas1. Interestingly, genes including Cxcl2 and Alc38a1 showed exclusive expression in only one or two cell type, pointing out the highly specialized functions of these genes in the context of AKI and ferroptosis.

### Heterogeneity of ferroptosis-related genes in proximal tubule cells

**Fig. 3:**
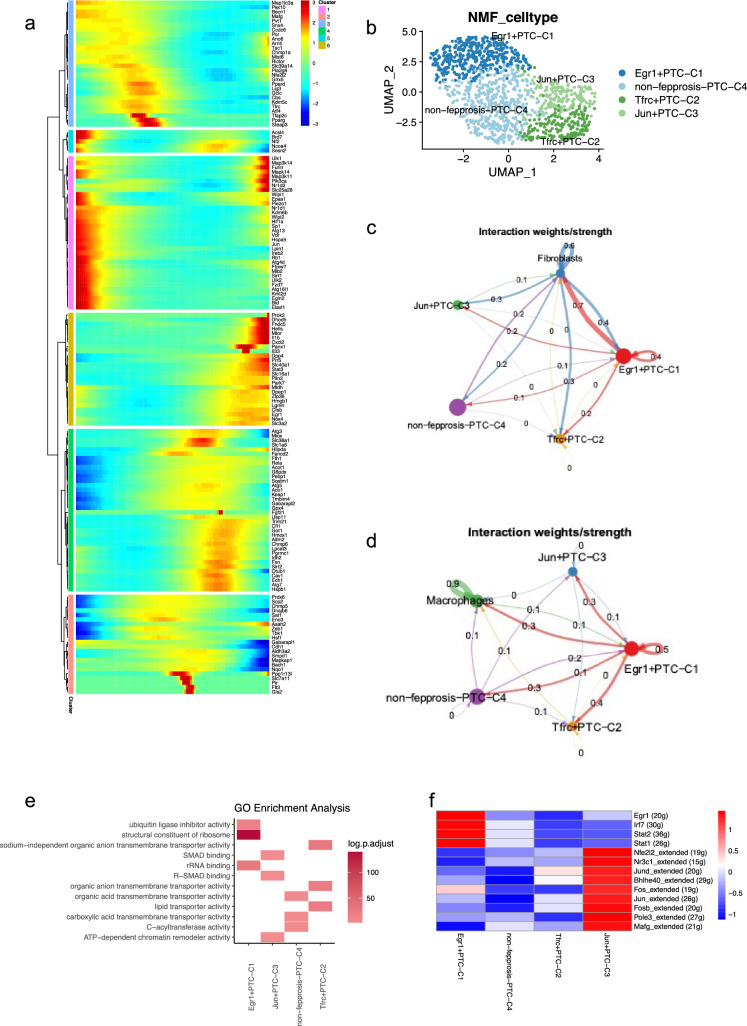
Proximal tubule cells show heterogeneity in the process and outcome of ferroptosis. **a** Pseudo-time analysis of the ferroptosis-related genes in proximal tubule cells. Genes present within the pink module are predominantly expressed during the early stages of proximal tubule cell development, whereas genes within the earthy yellow module show higher expression levels during the later stages of proximal tubule cell development. **b** NMF analysis based on ferroptosis-related genes distinguished proximal renal tubular cells into 4 clusters. **c**, **d** Show the weights of the crosstalk pathways among all clusters of proximal tubule cells and fibroblasts, as well as macrophages. **e** Significant pathways obtained by GO enrichment analysis of the highly expressed genes in the proximal tubule cells of each cluster. **f** The heatmap of the transcription factors activity of each proximal tubule cell cluster obtained by SENIC analysis

Through pseudo-time analysis, we discovered substantial heterogeneity in the expression of ferroptosis-related genes at different stages of proximal tubule cell. This suggested the necessity of conducting more refined clustering based on ferroptosis-related genes. Genes such as Wipi1, Hspa5 and Jun reached peak expression abundance in proximal tubule at early stage. Conversely, genes like Dhodh and Il1b were found to show significantly higher expression abundance at later stages (Fig. 3a). The heatmap generated from the pseudo-time analysis revealed that various ferroptosis-related genes were present at different developmental stages of proximal tubule cells. The outcome of this study emphasized the importance of performing a more in-depth clustering approach to better understand the complex heterogeneity of ferroptosis-related gene expression in proximal tubule cells, which can provide insights into their roles across cellular stages. Moreover, kidney injury markers based on previous studies further verified the authenticity of the pseudo-time analysis. Havcr1 (also known as Kidney Injury Molecule 1, Kim1) is commonly regarded as a sensitive and specific biomarker for kidney injury, exhibiting significantly increasing expression during the damage of renal tubules. It was found that the expression of Havcr1 did progressively increase over time (Supplementary 1a, c) [37]. Similarly, Neutrophil Gelatinase-Associated Lipocalin (lipocalin 2, Lcn2), also known as Ngal, a widely recognized biomarker for kidney injury, demonstrated a parallel increasing trend with Havcr1 (Supplementary 1b, c) [38]. Building on this foundation, we utilized NMF analysis and defined 4 clusters of proximal tubule cells. As detailed in the Methods section, 4 cell groups were distinguished by NMF, where cluster 1 was characterized by Egr1, cluster 2 was characterized by Tfrc, cluster 3 was characterized by Jun and cluster 4 has no characterized gene. Therefore, Egr1 + PTC-C1, Tfrc + PTC-C2, Jun + PTC-C3 and non-ferroptosis PTC-C4, were defined (Fig. 3b). This nuanced classification through NMF analysis allowed for a more refined understanding of the diversity within proximal tubule cells, especially in the context of ferroptosis.

Using the analysis of the Cellchat package, we found out that Egr1 + PTC-C1 interacted significantly more pronounced with fibroblasts and macrophages than all the remaining 3 proximal tubule cell types (Fig. 3c, d). Further exploration through GO enrichment analysis showed different pathway activities within the 4 clusters of proximal tubule cells. Egr1 + PTC-C1 cells displayed increased activities in the structural constituent of the ribosome, ubiquitin ligase inhibitor activity as well as rRNA binding pathways, which might indicate that protein synthesis and metabolism were at a higher level in this cluster of cells, while ATP-dependent chromatin remodeler activity, R-SMAD binding, and SMAD binding pathways were increased in Jun + PTC-C3 cells, reflecting the active histone modification in this cluster of cells. Sodium independent organic anion transmembrane transporter activity, organic anion transmembrane transporter activity and lipid transporter activity in Tfrc + PTC-C2 were notably high. Meanwhile, organic acid transmembrane transporter activity, carboxylic acid transmembrane transporter activity and C-cyltransferase activity showed significant enrichment in non-ferroptosis PTC-C4 (Fig. 3e).

The heatmap of SENIC analysis reveals that the transcription factors in the Egr1 + PTC-C1 and Jun + PTC-C3 clusters were more active than in the other 2 clusters, including Tfrc + PTC-C2 and non-ferroptosis-PTC-C4. Egr1, Irf7, Stat2 and Stat1 were significantly more active in Egr1 + PTC-C1, while the Jun and Jund transcription factor binding sites were significantly more active in Jun + PTC-C3 (Fig. 3f).

Recent scholarly articles have identified Egr1 + PTC-C1, Jun + PTC-C3, and non-ferroptosis PTC-C4 as novel subgroups, implying that these profiles provide latest information about the kidney's cellular landscape (Supplementary 1d). The Tfrc + PTC-C2 cluster, on the other hand, might be like healthy proximal tubule cells previously characterized in the literature, indicating a baseline or reference state for comparison [39].

### Different metabolic and chemotactic orientations in macrophages during ferroptosis

**Fig. 4:**
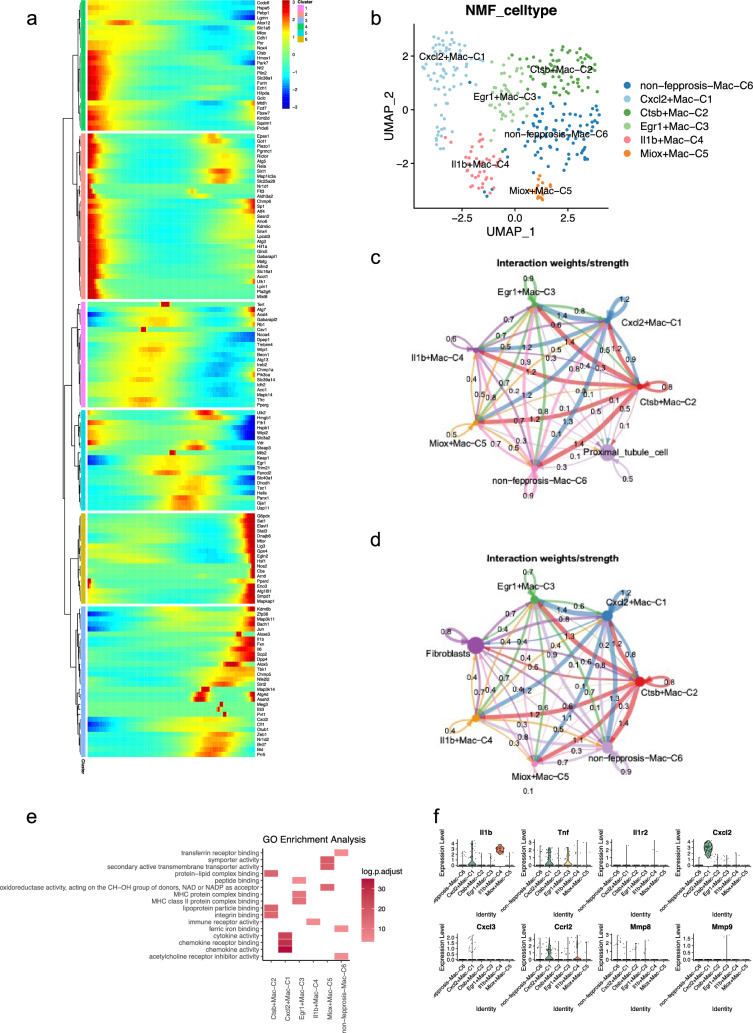
Macrophages exhibit different metabolic and chemotactic orientations during ferroptosis. **a** Pseudo-time analysis of the ferroptosis-related genes in macrophages. The genes within the red module are more expressed in the early stage of macrophage development, while the genes of the earthy yellow and blue modules are more expressed in the late stage of macrophage development. **b** NMF analysis based on ferroptosis-related genes distinguished macrophages into 4 clusters. **c**, **d** show the weights of the crosstalk pathways among all clusters of macrophages and proximal tubule cells, as well as fibroblasts. **e** Significant pathways obtained by GO enrichment analysis of the highly expressed genes in the macrophages of each cluster. **f** Violin plots of inflammatory and immune-related genes expressed brightly in each cluster of macrophages

The pseudo-time analysis heatmap revealed that different ferroptosis-related genes were expressed at different cellular stages of macrophages, suggesting the need for a deeper clustering of the heterogeneity of ferroptosis-related genes in macrophages (Fig. 4a). Expression of genes, including Ctsb, Hmox1, Park7, Epas1 and so on, were highest in early status, while expression of genes such as Il1b, Kdm6b, Bach1 and Jun peaked in late ones.

We used NMF clustering analysis to categorize AKI mice kidney macrophages, we successfully differentiated six distinct clusters. These clusters were Cxcl2 + Mac-C1, Ctsb + Mac-C2, Egr1 + Mac-C3, Il1b + Mac-C4, Miox + Mac-C5, and non-ferroptosis-Mac-C6 (Fig. 4b). The NMF analysis facilitated the segregation of six macrophage clusters in AKI mice kidneys, each of them defined by distinct gene expression patterns. Cluster 1 exhibited high expression of Cxcl2, cluster 2 was characterized by elevated levels of Ctsb, cluster 3 demonstrated significant expression of Egr1, cluster 4 displayed notable expression of Il1b, cluster 5 showed elevated expression of Miox, whereas cluster 6 did not exhibit any specific gene characterization. Cellchat analysis showed that Cxcl2 + Mac-C1 and Ctsb + Mac-C2 were the most communicative, showing the highest levels of interactions with each of the other clusters of macrophages (Fig. 4c, d). Specifically, we have found out that Ctsb + Mac-C2 had the highest Interaction weight with proximal tubule cells compared to the other 5 clusters of macrophages (Fig. 4c). Interactions between Cxcl2 + Mac-C1 and Egr1 + Mac-C3 were the most potent with fibroblasts (Fig. 4d).

GO enrichment analysis showed that lipid metabolism-related pathways, including integrin binding, lipoprotein particle binding and protein lipid complex binding, were prominently more active in Ctsb + Mac-C2. This suggested a significant role in lipid processing and metabolism within this cluster. In contrast, the pathways of chemokine activity, chemokine receptor binding and cytokine activity were more active in Cxcl2 + Mac C1, hence indicating an elevated capacity for initiating and propagating inflammatory response and chemotaxis. Additionally, pathways related to peptide binding, MHC class II protein complex binding and MHC protein complex binding were enhanced in Egr1 + Mac-C3. This enhancement suggested a pivotal role in regulating T cell-mediated immune responses, potentially influencing the overall immune landscape within the AKI context. In Miox + Mac-C5, we found that the symporter activity and secondary active transmembrane transporter activity pathways were significantly more enriched, suggesting that the cluster of cells was exhibiting a higher level of activity in the transmembrane transport of substances, indicating an enhanced capacity for moving materials across cell membranes. Moreover, immune receptor activity was observed to be elevated, indicating an enhanced responsiveness of the immune system in Il1b + Mac − C4, while transferrin receptor binding, ferric iron binding and acetylcholine receptor inhibitor activity were enhanced in non-ferroptosis-Mac-C6 (Fig. 4e).

Violin plot analysis of the inflammatory and immune-related genes revealed that the expression of inflammatory response-related genes Il1b, Tnf, Cxcl2, Cxcl3 and Ccrl2 were highest in Cxcl2 + Mac-C1. An analysis that further supported the conclusion that this cluster of cells exhibited the most potent inflammatory response and chemotactic activity among the six identified macrophage clusters (Fig. 4f).

### Ferroptosis-related genes distinguished the heterogeneity of fibroblasts

**Fig. 5:**
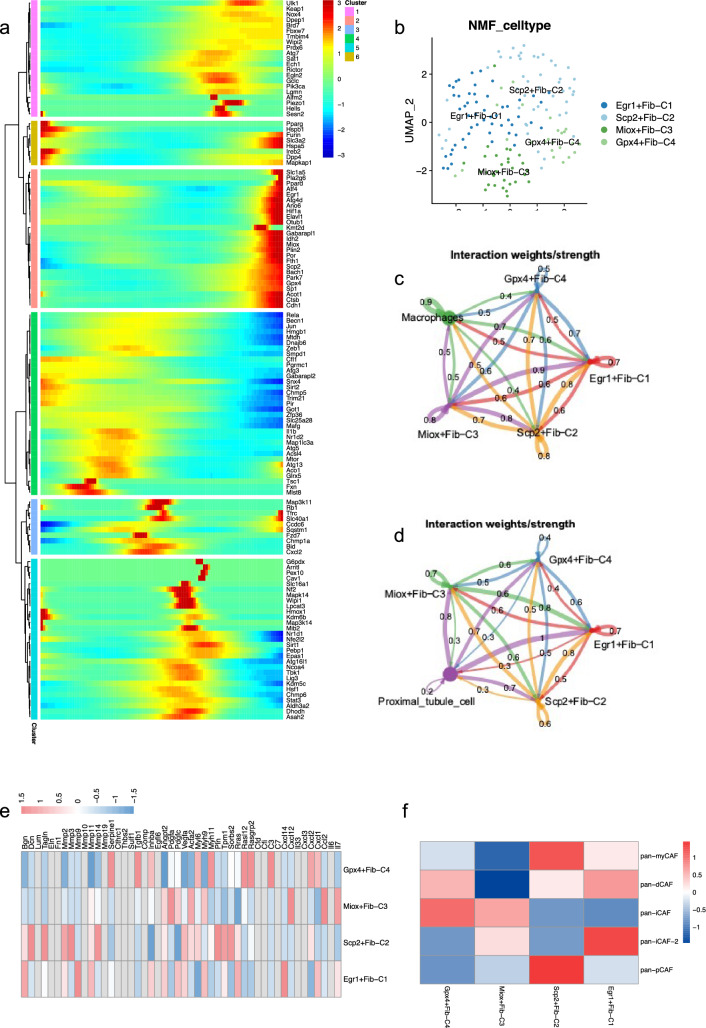
Ferroptosis-related genes distinguished the heterogeneity of fibroblasts. **a** Pseudo-time analysis of the ferroptosis-related genes in fibroblasts. The genes within the earth-yellow module are expressed more in the initial stages of fibroblast development, while the red genes are expressed more in the late stages of fibroblast development. **b** NMF analysis based on ferroptosis-related genes distinguished macrophages into 4 clusters. **c**, **d** show the weights of the crosstalk pathways among all clusters of fibroblasts and macrophages, as well as proximal tubule cells. **e** Assessment of the immune microenvironment of each cluster of fibroblasts. **f** Heatmap of CAF signature score for each cluster of fibroblasts

Pseudo-time analysis of ferroptosis-related gene expression in fibroblasts revealed that fibroblast also displayed time-dependent heterogeneity (Fig. 5a). Dpp4 had the highest expression in early fibroblast status, while Gpx4, Scp2 and Egr1 spiked in late fibroblast status. The pseudo-time analysis heatmap, revealed that different fibroblasts exhibit the presence of various ferroptosis-related genes at different stages. This observation highlighted the importance of conducting a more comprehensive clustering analysis paving the way for a better understanding of the heterogeneity of ferroptosis-related genes in fibroblasts.

NMF analysis was employed to cluster fibroblasts into four distinct groups, namely Egr1 + Fib-C1, Scp2 + Fib-C2, Miox + Fib-C3, and Gpx4 + Fib-C4 (Fig. 5b). We used NMF clustering analysis to categorize AKI mice kidney macrophages into these four distinctive clusters: Egr1 + Fib-C1, Scp2 + Fib-C2, Miox + Fib-C3, and Gpx4 + Fib-C4. By analyzing specific gene expression profiles, the NMF analysis facilitated the differentiation and classification of these four cell groups, providing a clear delineation based on their unique molecular characteristics. Cluster 1 showed an elevated expression of Egr1, cluster 2 was characterized by increased levels of Scp2, cluster 3 demonstrated significant expression of Miox, and cluster 4 displayed notable expression of Gpx4. CellChat analysis showed that Egr1 + Fib-C1 had the strongest intercellular interactions with both proximal tubule cells and macrophages (Fig. 5c, d).

Heatmap analysis detailing the expression of extracellular matrix metabolism-related genes showed that Egr1 + Fib-C1 had the highest expression of Bgn, Mmp9 and Cxcl14. Additionally, Scp2 + Fib-C2 showed significantly higher expressions of Dcn, Ragln, Mmp3, and Pln, whereas Miox + Fib-C3 showed elevated levels of Pdgfa, Cxcl12, Ccl2, and Il7, indicating distinct functional emphases within these fibroblast subsets. While the expression abundance of Serpine1, Tgfb1, Myh11, Rasl12, Rasgrp2 and C3 was higher in Gpx4 + Fib-C4 as presented in Fig. 5e.

Correlation analysis with tumor-associated fibroblasts showed that Egr1 + FibC1 and Scp2 + Fib-C2 were highly correlated with pan-iCAF-2 and pan-pCAF, respectively (Fig. 5f). The analysis revealed that Egr1 + Fib-C1 showed a negative correlation with pan-pCAF and pan-iCAF. Among them, Pan-pCAF was associated with cell proliferation, while Pan-iCAF was thought to control the transcriptional program associated with inflammation [40]. Some studies have reported the association of pan-iCAF-2 with extracellular matrix remodeling, while pan-pCAF was associated with regulation of cell proliferation [40].

According to the most recent studies, the four identified fibroblast subgroups had characteristics like previously reported mesangial cells (Supplementary 1e). This finding implied that mesangial cells played a critical role in the onset of ferroptosis during acute kidney injury, emphasizing their importance in the pathological process [39].

### Validation of heterogeneity of ferroptosis-related genes in AKI at the RNA-seq level

**Fig. 6:**
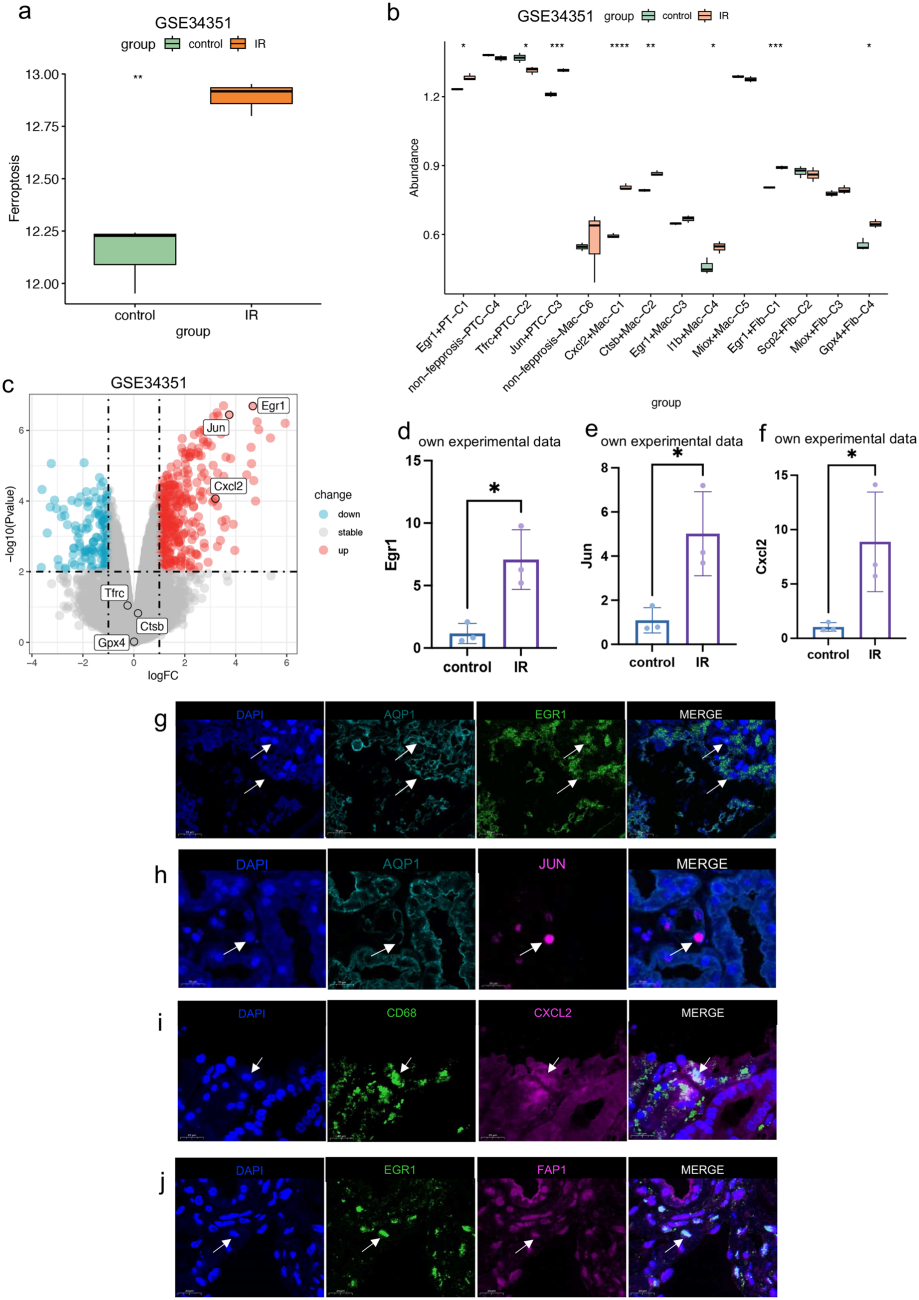
Transcriptomic validation of study results by GSE34351 and in vivo models. **a** Expression levels of ferroptosis-related genes in control (*n* = 3) and IR model mice (*n* = 3). **b** Infiltration abundance of characteristic genes of each NMF typing cell cluster in control and IRI mice model. **c** Volcano maps of DEG analysis between control and IRI mice model with annotation of cell cluster marker genes with significantly different abundance of characteristic gene infiltration. **d** PCR results of Egr1 transcript levels in an in vivo model (*n* = 3 vs *n* = 3). **e** PCR results of Jun transcript levels in an in vivo model (*n* = 3 vs *n* = 3). **f** PCR results of Cxcl2 transcript levels in an in vivo model (*n* = 3 vs *n* = 3). **g** Colocalization of AQP1 and Egr1 confirming the presence of Egr1 + PTC-C1. **h** Colocalization of AQP1 and Jun confirming the presence of Jun + PTC-C3. **i** Colocalization of CD68 and Cxcl2 confirming the presence of Jun + PTC-Cxcl2 + Mac-C1. **j** Colocalization of FAP and Egr1 confirming the presence of Egr1 + Fib-C1

Analysis of GSE34351 demonstrated that the collective expression of 205 ferroptosis-related genes was notably elevated in the kidneys of the AKI mouse model in comparison with the control samples. We extracted the characteristic genes of each cluster of cells and analyzed the infiltration in the kidneys of IRI mice model and controls (Fig. 6b). A total of 8 clusters of cells, including Egr1 + PTC-C1, Tfrc + PTC-C2, Jun + PTC-C3, Cxcl2 + Mac-C1, Ctsb + Mac-C2, l1b + Mac-C4, Egr1 + Fib-C1, and Gpx4 + Fib-C4, had significant differences in the abundance of their signature genes infiltrating the kidneys between AKI and control mice.

The expression of the 7 signature genes across the 8 clusters of cells, namely Egr1, Tfrc, Jun, Cxcl2, Ctsb, l1b, and Gpx4, were validated in the kidneys of IRI mice and controls, and revealed that Egr1, Jun and Cxcl2 expression was significantly higher in AKI mice (Fig. 6c). RT-qPCR of Egr1, Jun and Cxcl2 from kidney revealed that all the 3 genes were significantly elevated in IRI mice compared to controls (Fig. 6d–f).

Multiplex immunofluorescence staining on renal pathology biopsy samples from human AKI patients was performed. This staining confirmed the presence of Egr1 + PTC-C1, Jun + PTC-C3, Cxcl2 + Mac-C1, and Egr1 + Fib-C1 cells during the occurrence of AKI. This technique demonstrated fluorescence co-localization, providing clear visual evidence of these specific cell types during AKI episodes. We used AQP1 immunofluorescence to localize proximal tubule cells and observed their co-staining with Egr1 and Jun, confirming the presence of Egr1 + PTC-C1 (Fig. 6g) and Jun + PTC-C3 (Fig. 6h). Similarly, we used CD68 to locate macrophages and observed their fluorescence colocalization with Cxcl2, confirming the presence of Cxcl2 + Mac-C1 (Fig. 6i). Likewise, we used FAP to locate fibroblasts and observed their co-staining with Egr1, confirming the presence of Egr1 + Fib-C1 (Fig. 6j).

## Discussion

An increasing amount of evidence concurs with the vital function of ferroptosis in the initial stages and progression of AKI. Despite this, the specific cellular mechanisms and diversity of ferroptosis within the AKI microenvironment remain unclear, particularly at the single-cell level. In this study, we discovered the heterogenous expression of ferroptosis-related genes at various times of cell status in proximal tubule cells, macrophages, and fibroblasts. A total number of 14 clusters of the 3 cell categories were identified by NMF analysis and cell-to-cell interactions were analyzed. Using a combined GO enrichment analysis, SCENIC analysis, and correlation analysis, we re-examined the underlying pathways responsible for the differential distribution of cell clusters. This reanalysis focused on cell-to-cell communication, cytokine expression, and inflammatory infiltration, offering deeper insights into the complex interactions within the cellular landscape. By screening the 14 clusters of cells in the transcriptomic RNA-seq dataset, 8 clusters had significant differences in the abundance of their signature genes in kidney between AKI and control mice, including Egr1 + PT-C1, Tfrc + PTC-C2, Jun + PTC-C3, Cxcl2 + Mac-C1, Ctsb + Mac-C2, l1b + Mac-C4, Egr1 + Fib-C1, and Gpx4 + Fib-C4. The expressions of Egr1, Jun and Cxcl2 were significantly higher in IRI mice compared to controls, therefore validating our findings in vivo.

Utilizing CellChat analysis, we uncovered the intricate crosstalk occurring between subsets of proximal tubular cells, macrophages, and fibroblasts. First, Egr1 + PTC-C1 interacted more significantly with fibroblasts and macrophages. Increased structural constituent of ribosome, ubiquitin ligase inhibitor activity, and rRNA binding pathway activity in Egr1 + PTC-C1 cells, which might indicate that protein synthesis and metabolism were at a higher level in this cluster of cells [41, 42].

Second, Egr1 + Fib-C1 had the strongest intercellular interactions with both proximal tubule cells and macrophages. The heatmap of the expression of extracellular matrix metabolism-related genes showed that Egr1 + Fib-C1 had the highest expression of Bgn, Mmp9 and Cxcl14 [43, 44].

Third, Ctsb + Mac-C2 had shown the highest Interaction weight with proximal tubule cells, while Cxcl2 + Mac-C1 and Egr1 + Mac-C3 were the most potent with fibroblasts. Lipid metabolism-related pathways, including integrin binding, lipoprotein particle binding and protein lipid complex binding, were more active in Ctsb + Mac-C2 [45, 46]. Pathways of chemokine activity, chemokine receptor binding and cytokine activity were more active in Cxcl2 + Mac C1, implying an enhanced inflammatory response and chemotactic function [47, 48]. Peptide binding, MHC class II protein complex binding and MHC protein complex binding are enhanced in Egr1 + Mac-C3, which might play a regulatory role on the immune response of T cells [49, 50].

By exploring more thoroughly into the transcriptomic RNA-seq dataset with the 14 identified cell clusters, we isolated eight clusters that exhibited greater levels of expression of signature genes in the kidneys of AKI mice compared to control samples. Among the 8 clusters, 7 signature genes were extracted and 3 of them stood out to be significantly elevated in AKI mice, including Egr1, Jun and Cxcl2. Egr1 + PTC and Egr1 + Fibroblast were higher in abundance in AKI mice kidneys compared with controls. In addition, in cell crosstalk, Egr1 + PTC-C1 interacted more significantly with fibroblasts and macrophages, as well as Egr1 + Fib-C1. Early growth response 1 (EGR1) is a transcription factor that plays a critical role in regulating various cellular processes, including cell differentiation, apoptosis, and inflammation [51, 52]. Recent studies have suggested that EGR1 may also be involved in the pathogenesis of AKI [53, 54]. In different animal models of AKI. EGR1 has been demonstrated to elevate SOX9 expression in renal tubular cells. EGR1 interacts directly with the Sox9 gene promoter, stimulating the development of SOX9-positive cells by activating the Wnt/β-catenin signaling pathway [53]. PPARγ/EGR1 pathway could also suppressing NF-κB mediated inflammation in AKI [55]. Our research discovered that Egr1 + PTC owned stronger communications with other cells in the microenvironment, serving as catalysts for cell proliferation and immune inhibition. Based on these finding we speculated that Egr1 + PTC might play a protective factor in AKI and EGR1 could be a potential therapeutic target for AKI [56]. Despite the encouraging potential of Egr1 + PTC in AKI, more research is needed to understand the precise mechanisms by which it functions. These additional studies will provide further insight on the role of Egr1 + PTC in AKI pathology, as well as aid in determining its potential clinical significance and utility.

JUN is a member of the activator protein-1 (AP-1) transcription factor family transcription factor that has been implicated in the pathogenesis of AKI [57]. Studies have shown that JUN plays a critical role in the development of AKI by regulating various cellular processes such as apoptosis, inflammation, and oxidative stress [58]. A decrease of Jun protected renal ischemia–reperfusion injury-induced apoptosis and sepsis-induced AKI by inhibiting inflammation and oxidative stress [59–61]. Interestingly, the KEGG enrichment in our study revealed that ATP dependent chromatin remodeler activity was upregulated specifically in Jun + PTC. These findings suggested that Jun could be an appealing target for AKI treatment, requiring further investigation into inhibition strategies. Of interest is that for proximal tubule cells, the ferroptosis-featured cell subpopulations Egr1 + PTC and Jun + PTC were both characterized by transcription factors and the two transcription factors led to opposite functions, suggesting that core transcription factors might regulate PTCs and determine different directions of cell differentiation [62, 63]. Combating the pressing need for additional research, strategies to regulate the balance between the two differentiation directions and steer cells toward Egr1 + proximal tubular cells (PTC) to mitigate the negative prognosis of AKI via ferroptosis remain a critical challenge.

CXCL2, also known as macrophage inflammatory protein-2 (MIP-2), is a chemokine that plays a critical role in the pathogenesis of various inflammatory-related diseases. For ferroptosis, it has been reported that CXCL2 inhibited cell growth and could improve cellular ROS and Fe2 + levels, resulting in ferroptosis cell death [64]. Recent researches discovered that upregulation of CXCL2 contributed to the recruitment and activation of neutrophils, which played a key role in the development of AKI [65]. Thus, CXCL2 impacted AKI in both ferroptosis pattern and chemotaxis. Knockout of CXCR2, which is the receptor of CXCL2 could protect mice against DSS-colitis-induced AKI and inflammation, indicating the therapeutic value of CXCL2 in AKI [66]. Additionally, as a secreted protein, CXCL2 could also serve as a diagnostic and prognostic biomarker for AKI by urine testing [67]. In addition, our research indicated that macrophages were the main culprit for the elevated levels of CXCL2 in AKI. These CXCL2-expressing macrophages exhibited distinct chemokine and cytokine activities, which coincided with ferroptosis. However, limited to the easy loss of neutrophils in single-cell sequencing, we were unable to obtain evidence of macrophage-neutrophil communication at this single-cell level, which needed to be further validation in future high-quality single-cell sequencing.

It is important to note that the subgroups we have defined, Egr1 + PTC-C1, Jun + PTC-C3, and non-ferroptosis PTC-C4, are novel subsets of PTC. On the other hand, Tfrc + PTC-C2 may be like the previously reported healthy PT. In fact, our findings were validated both at the transcriptomic level and through in vivo experiments. Marker genes Egr1 and Jun showed a significant upregulation in the kidneys of AKI mice, while Tfrc did not exhibit significant changes in subsequent analyses. This further suggested that Tfrc + PTC-C2 might be close to the already discovered healthy PT subset [39].

Additionally, drawing from recent literature, our research has delineated four distinct subgroups of fibroblasts: Egr1 + Fib-C1, Scp2 + Fib-C2, Miox + Fib-C3, and Gpx4 + Fib-C4. These subgroups shared similarities with the previously reported mesangial cells. These findings indicated that mesangial cells could play a pivotal role as the primary fibroblast population involved in iron-induced cell death during AKI [39].

Unquestionably, our study possessed several strengths, along with some limitations. First, we defined several novel proximal tubular cells, fibroblasts and macrophages from single cell analysis that might play a significant role in the development of AKI. Second, we further validated the results of single-cell analysis by in-depth transcriptomic Bulk analysis and finally, we validated the results of single-cell analysis and Bulk analysis in animal models. However, due to constraints in current experimental conditions, co-culturing three types of cells—proximal tubule cells, fibroblasts, and macrophages—was not possible. As a result, the findings of single-cell analysis could not be validated using an in vitro assay. Therefore, we conducted multiplex immunofluorescence staining on renal pathology biopsy samples from human AKI patients. This staining confirmed the presence of Egr1 + PTC-C1, Jun + PTC-C3, Cxcl2 + Mac-C1, and Egr1 + Fib-C1 cells during the occurrence of AKI.

## Conclusion

In conclusion, we provided a profound analysis of ferroptosis among various cell types in the AKI kidney microenvironment at the single-cell level in this study. We also identified a series of novel cell subtypes with heterogeneous ferroptosis patterns, including EGR1 + PTC, JUN + PTC and CXCL2 + macrophages, and validated them using an IR animal model.

In this research, we have identified new cell subtypes, including Egr1 + PTC-C1, Jun + PTC-C3, Cxcl2 + Mac-C1 and Egr1 + Fib-C1. These cell clusters play distinct roles in the kidneys of AKI mice compared to normal mice. Most importantly, the novel clusters of cells we have identified and the crosstalk between them can suggest mechanisms for the development of AKI. By blocking or promoting the development of a cluster of cells or crosstalk with other cells has the potential to be a new target for the clinical treatment of AKI. The expressions of Egr1, Jun, and Cxcl2 were higher in the IRI mice model than controls. Multiplex immunofluorescence staining on renal pathology biopsy samples from human AKI patients further confirmed the presence of Egr1 + PTC-C1, Jun + PTC-C3, Cxcl2 + Mac-C1, and Egr1 + Fib-C1 cells during the occurrence of AKI. In conclusion, our study provides a pioneering analysis of the intricate cell-to-cell interactions that occur during AKI, with an especially heavy focus on the mechanism of ferroptosis.

### Supplementary Information

Below is the link to the electronic supplementary material.

Supplementary file1 (PDF 359 KB)

## Data Availability

Public data can be obtained from GEO (https://www.ncbi.nlm.nih.gov/geo/) database. Other queries for data and code could be directly sent to the corresponding author.
