# Supplementary figures and images for: Crosstalk among proximal tubular cells, macrophages, and fibroblasts in acute kidney injury: single-cell profiling from the perspective of ferroptosis
Source: Hum Cell. 2024 May 16;37(4):1039–55. doi: 10.1007/s13577-024-01072-z (PMC11194220; doi:10.1007/s13577-024-01072-z)

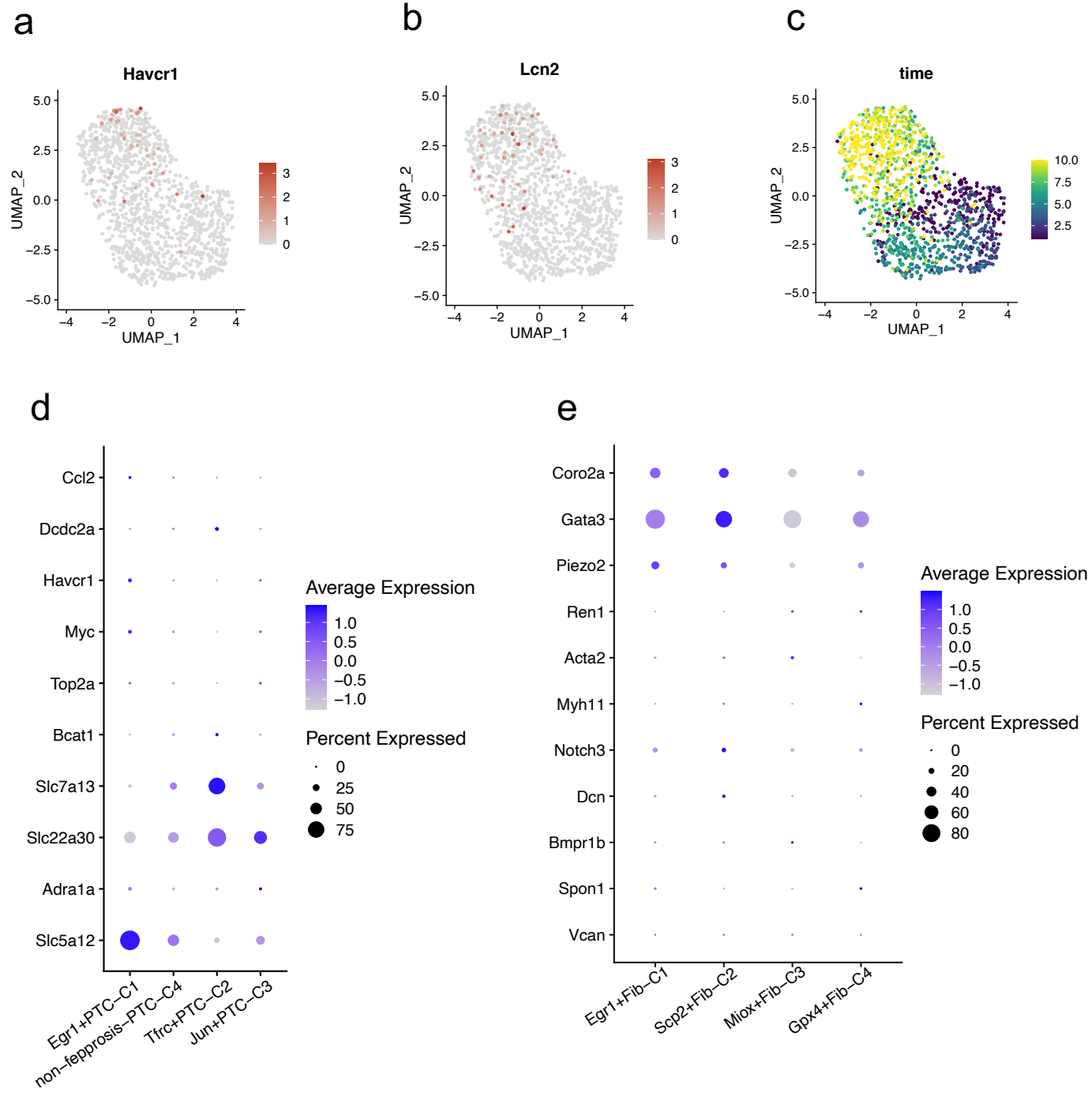

Supplementary 1

Supplement: Supplementary file 1 — Supplementary file1 (PDF 359 KB) [file 13577_2024_1072_MOESM1_ESM.pdf]
